# Saireito (TJ-114) for Preventing High-Output Syndrome After Temporary Ileostomy in Rectal Cancer Surgery

**DOI:** 10.1007/s00384-025-04983-x

**Published:** 2025-10-06

**Authors:** Mamoru Uemura, Chikako Kusunoki, Yuki Sekido, Mitsunobu Takeda, Tsuyoshi Hata, Atsushi Hamabe, Takayuki Ogino, Norikatsu Miyoshi, Mitsuyoshi Tei, Yoshinori Kagawa, Hidetoshi Eguchi, Yuichiro Doki

**Affiliations:** 1 Department of Gastroenterological Surgery, Graduate School of Medicine, The University of Osaka, 2-2 Yamada-Oka, Suita City, Osaka 565-0871 Japan; 2 Department of Surgery, Osaka Rosai Hospital, Sakai, Japan; 3 Department of Gastroenterological Surgery, Osaka International Cancer Institute, Osaka, Japan

**Keywords:** Rectal cancer, Temporary ileostomy, High-output syndrome, Kampo medicine, Saireito

## Abstract

**Purpose:**

In rectal cancer surgery, a temporary ileostomy is often created to prevent clinical anastomotic leakage. However, high-output syndrome (HOS) frequently arises as a postoperative complication. We aimed to investigate the preventive effect of Saireito, a traditional Japanese herbal medicine, on HOS in patients with a temporary ileostomy.

**Methods:**

At the University of Osaka Hospital, Saireito has been routinely administered to patients undergoing rectal cancer surgery with a temporary ileostomy since October 2022. Patients received 9 g/day of Saireito in three divided doses starting from the afternoon of postoperative day 1 (POD1). Among 68 consecutive patients, five were excluded due to incomplete administration of Saireito according to the planned postoperative schedule. The remaining 63 patients were included in the analysis and divided into two groups: the Saireito group (n = 37) and the control group (n = 26). HOS was defined as stoma output ≥ 1,500 mL/day by POD10.

**Results:**

There were no significant differences in patient background characteristics between the two groups (p > 0.05). HOS occurred in 13 patients (20.6%). The incidence of HOS was significantly lower in the Saireito group (4 / 37, 10.8%) compared with the control group (9 / 26, 34.6%) (p = 0.0291).

**Conclusion:**

Postoperative administration of Saireito in rectal cancer patients with temporary ileostomy was associated with a reduced incidence of high-output syndrome. Saireito may be a promising option for preventing HOS in such cases.

**Supplementary Information:**

The online version contains supplementary material available at 10.1007/s00384-025-04983-x.

## Introduction

In rectal cancer surgery, the incidence of anastomotic leakage following low anterior resection has been reported to be 9.7–15.9%, and the mortality rate associated with leakage is as high as 6–22% [1–4]. To reduce the rates of clinical leakage, reoperation, and mortality, temporary diverting stomas are frequently constructed. However, a temporary ileostomy can lead to high-output syndrome (HOS), a significant clinical issue. The reported incidence of HOS ranges from 16% to 43.3%, and it accounts for 4% to 43% of readmissions after surgery [4–8]. HOS is defined as a daily stoma output exceeding 1,500 mL, which can cause dehydration and electrolyte imbalance, often requiring fluid and electrolyte replacement as well as antisecretory or antidiarrheal agents [8, 9].

The perioperative use of Kampo medicines (traditional Japanese herbal medicines), which have relatively mild but multifaceted pharmacologic effects, in gastrointestinal surgery has gained increasing attention, with several studies suggesting their clinical utility [10]. Among these, Daikenchuto has been extensively investigated for its potential benefits in gastrointestinal surgery. It has been reported to suppress postoperative inflammatory responses—such as elevations in C-reactive protein [11]—and to reduce the incidence of postoperative ileus as well as promote faster recovery of bowel function after laparoscopic colorectal surgery [12].

Saireito, a Kampo formula composed of Shosaikoto and Goreisan, is officially approved under the national health insurance system in Japan and is commonly prescribed for conditions such as watery diarrhea, acute gastroenteritis, and edema [13, 14]. The detailed composition and quantities of the crude drugs in Saireito are specified in the manufacturer’s package insert (Tsumura Saireito Extract Granules for Ethical Use, TJ-114) in accordance with the Japanese Pharmacopoeia, and this formulation was used in the present study. The composition list, including English names, botanical names, and romanized Japanese names, is provided in the Supplementary materials (Supplementary Table 1). It exerts diuretic effects through sodium channel inhibition and also exhibits anti-inflammatory activity, suggesting potential regulation of fluid balance [15]. These properties raise the possibility that Saireito may help prevent HOS following rectal cancer surgery with temporary ileostomy.

The aim of this study was to investigate whether postoperative administration of Saireito reduces the incidence of HOS in patients who underwent rectal cancer surgery with the creation of a temporary ileostomy. This pilot study also serves as a basis for a forthcoming prospective randomized controlled trial to further evaluate the efficacy of Saireito in preventing HOS.

## Patients and methods

Between January 2022 and August 2024, we enrolled consecutive patients who underwent rectal cancer surgery with the simultaneous creation of a temporary ileostomy at our institution. Patients with underlying conditions considered likely to directly influence the development of HOS, such as inflammatory bowel diseases (Crohn’s disease or ulcerative colitis), were excluded. During the study period, 74 patients underwent rectal cancer surgery with creation of a temporary ileostomy. Of these, five patients had ulcerative colitis–associated colorectal cancer and underwent total colectomy, and one patient had recurrent fistula-associated carcinoma secondary to Crohn’s disease treated with total pelvic exenteration. These six cases met the predefined exclusion criteria and were therefore excluded. A total of 68 patients were included in the final analysis.

In October 2022, our department introduced the routine administration of Saireito as part of standard postoperative care. Saireito was thereafter administered to all patients undergoing rectal cancer surgery with a temporary ileostomy. Accordingly, patients who underwent surgery after this date were uniformly managed with postoperative Saireito administration, and those treated before this date served as the control group. Group assignment was therefore based solely on the date of surgery, without any patient- or surgery-related selection bias. In addition, because the study involved a relatively short consecutive series, perioperative management—including surgical procedures, fluid therapy, and postoperative care—was consistent across all patients, except for the use of Saireito.

**Fig. 1:**
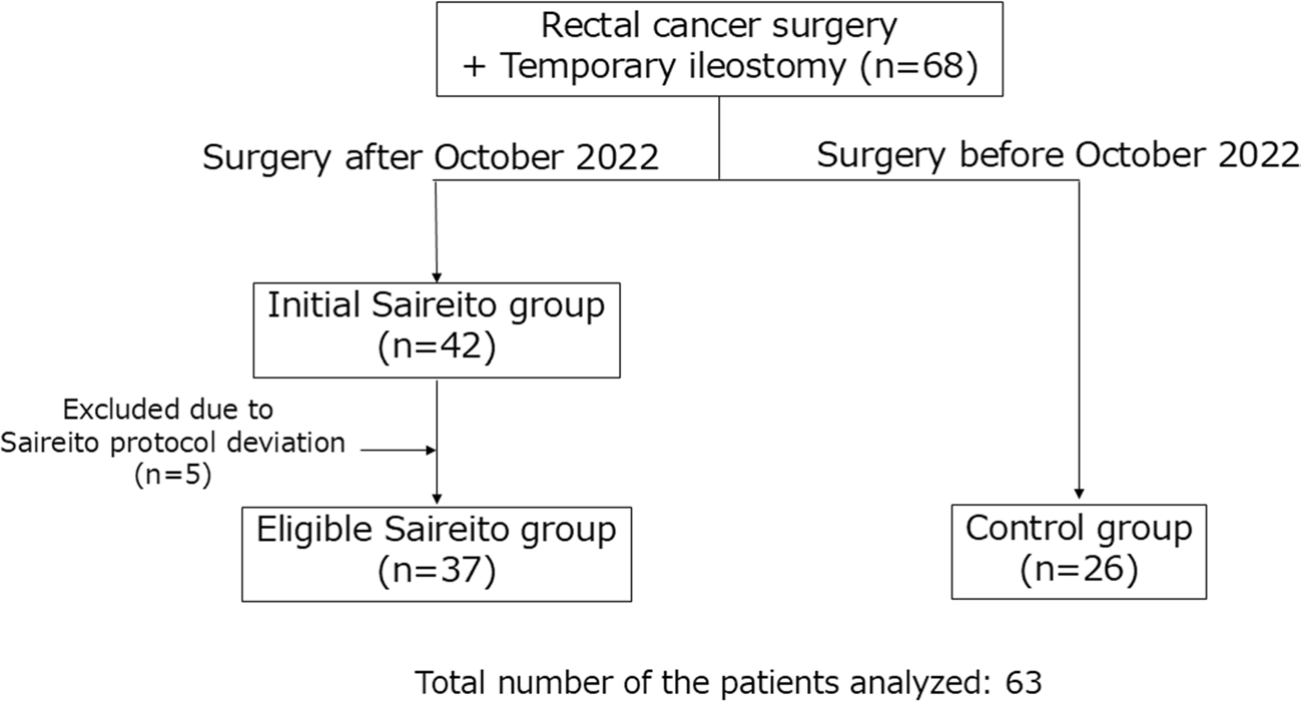
Flowchart of patient selection. Between January 2022 and August 2024, 68 patients underwent rectal cancer surgery with temporary ileostomy. After excluding those with inflammatory bowel disease and five patients who did not receive Saireito according to the scheduled protocol, 63 patients were included in the final analysis: 37 in the Saireito group and 26 in the control group

Saireito was administered orally in three divided doses per day (3 g per dose, totaling 9 g/day), starting from the afternoon of postoperative day 1 (POD1). Of the 42 patients treated after the introduction of routine Saireito use, five were excluded because they did not receive Saireito according to the scheduled protocol. The remaining 37 patients constituted the Saireito group, and the 26 patients treated before October 2022 constituted the control group (Fig. 1).

## Definition of high-output syndrome (HOS)

HOS was defined as a stoma output of ≥ 1,500 mL per day by postoperative day 10 (POD10). During hospitalization, stoma output was measured multiple times per day by ward nurses, and the total daily volume was calculated. In patients with high-volume output, the stoma appliance was directly connected to a drainage bag, and the total volume was measured from the bag. In other cases, effluent was collected from the stoma appliance and quantified using a graduated container. The measurements were recorded in the electronic medical records and used for this analysis. Because stoma output could only be accurately measured during hospitalization, and some patients were discharged after POD10, the analysis was limited to the early postoperative period. This study focused on evaluating the preventive effect of Saireito on early-onset HOS during hospitalization; therefore, late-onset HOS occurring after POD10 was not assessed.

## Statistical analysis

Numerical data are presented as medians with interquartile ranges (IQRs). Differences between the variables were compared using Fisher's exact test or chi-square test. Differences in quantitative parameters were compared using the Wilcoxon rank-sum test (Mann–Whitney U test). Cumulative survival curves were constructed with the Kaplan–Meier method. A *p*-value < 0.05 was considered to indicate statistically significant. Univariate and multivariate analyses were performed to identify factors associated with the development of HOS after surgery. All statistical analyses were carried out using JMP Pro software version 17.0.0 for Mac (SAS Institute Inc., Cary, NC, USA).

This study was approved by the institutional review board of The University of Osaka (Approval no. 20163–3). Informed consent was obtained from all participants in accordance with the institutional general consent policy for clinical research.

## Results

### Patients characteristics

A total of 68 patients underwent rectal cancer surgery with a temporary ileostomy during the study period. Of these, 42 patients underwent surgery after the introduction of routine postoperative Saireito administration. Among them, five patients were excluded because they did not follow the scheduled Saireito regimen. As a result, 37 patients were included in the Saireito group. The remaining 26 patients, who underwent surgery before the introduction of Saireito, comprised the control group. Saireito was continued throughout the hospital stay in all patients in the Saireito group, unless discontinued for clinical reasons.

There were no significant differences in baseline characteristics between the two groups, including age, sex, body mass index (BMI), tumor stage, surgical procedure, or laparoscopic surgery rate (all p > 0.05). However, the prevalence of smoking history was significantly lower in the Saireito group. The prevalence of diabetes mellitus also tended to be lower in the Saireito group, although the difference was not statistically significant (Table 1).

**Table 1:**
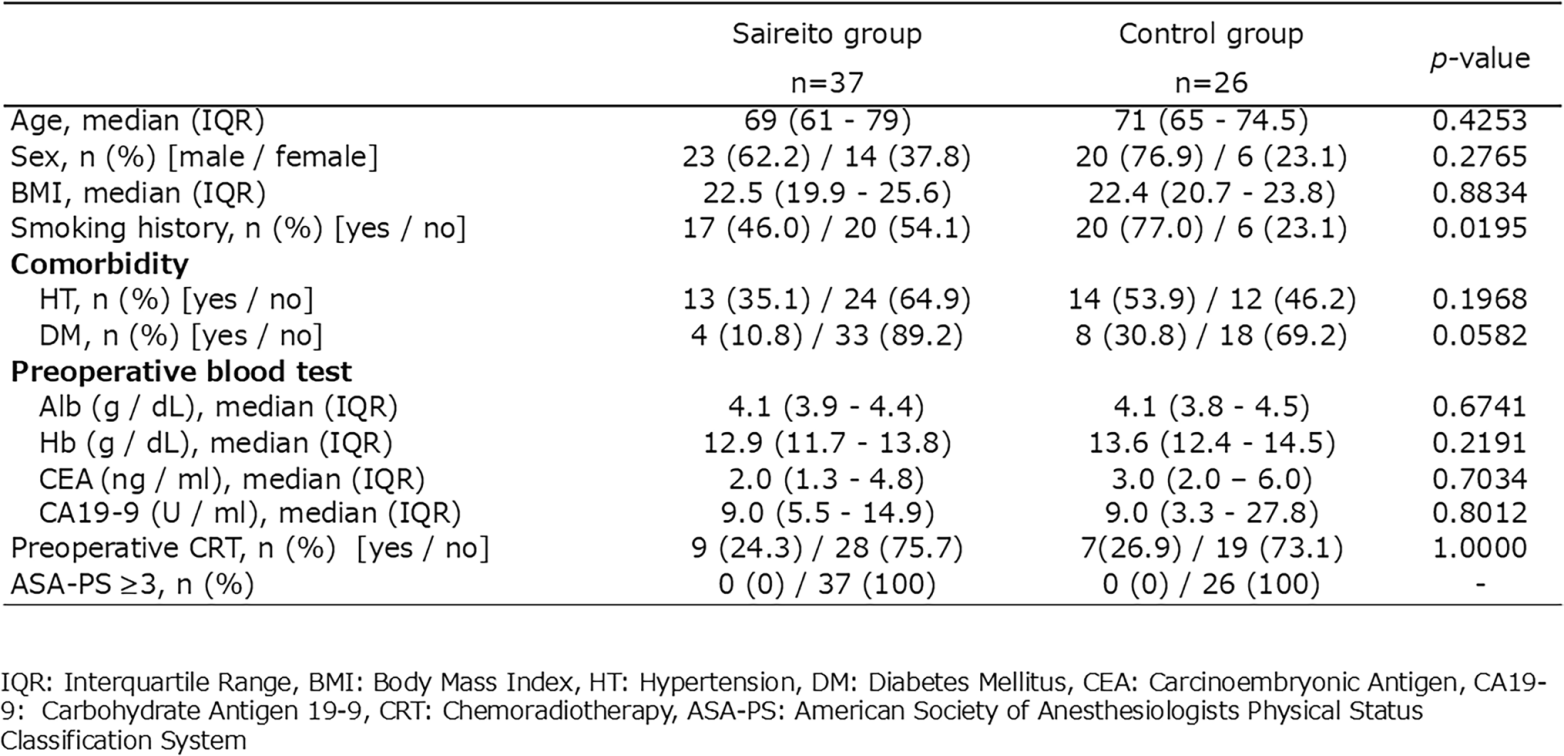
Baseline characteristics of the patients

### Surgical outcomes and postoperative course

A temporary ileostomy was constructed in all patients who underwent preoperative chemoradiotherapy. For other cases, a diverting stoma was created when the anastomosis was located within 4.5 cm from the anal verge.

There were no significant differences in surgical approach, anesthesia time, or blood loss between the two groups. Although not statistically significant, the use of loperamide was less frequent, and the length of hospital stay was slightly shorter in the Saireito group compared with the control group (Table 2).

**Table 2:**
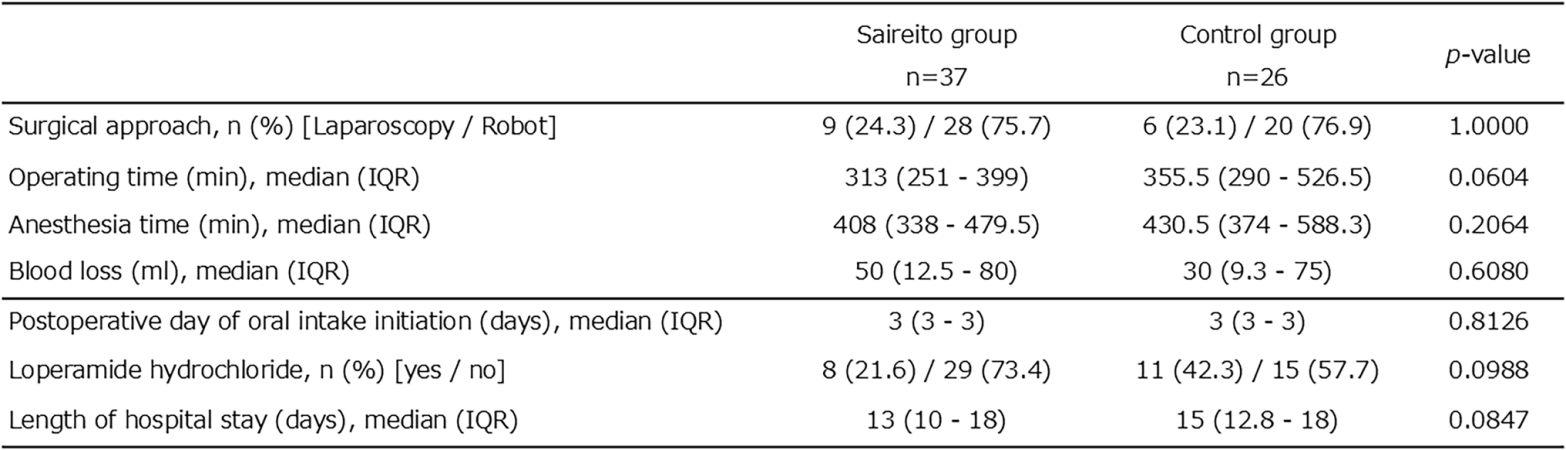
Perioperative and postoperative outcomes

### Incidence of high-output syndrome (HOS)

**Fig. 2:**
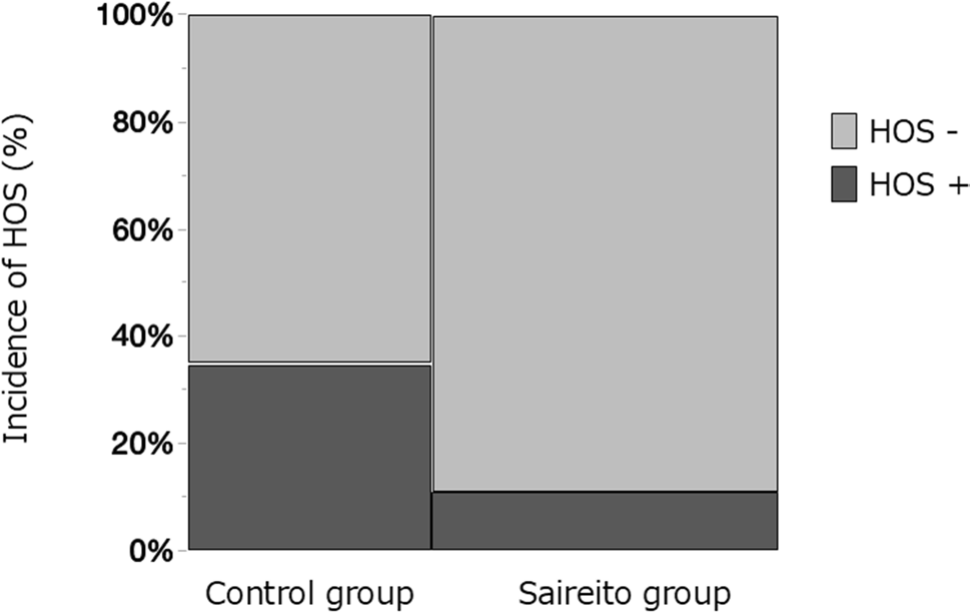
Incidence of high-output syndrome (HOS) in the Saireito and control groups. The incidence of HOS (defined as stoma output ≥ 1,500 mL/day by postoperative day 10) was significantly lower in the Saireito group (n = 37) compared to the control group (n = 26). Dark gray indicates HOS-positive cases; light gray indicates HOS-negative cases

High-output syndrome was observed in 13 patients overall (20.6%). The incidence was significantly lower in the Saireito group (4 of 37 patients, 10.8%) compared with the control group (9 of 26 patients, 34.6%) (p = 0.0291). The relative risk was 0.31 (95% CI, 0.11–0.90), indicating that the risk of HOS in the Saireito group was approximately one-third that of the control group. This difference was statistically significant, suggesting that postoperative administration of Saireito may contribute to the prevention of early-onset HOS (Table 3, Fig. 2).

**Table 3:**
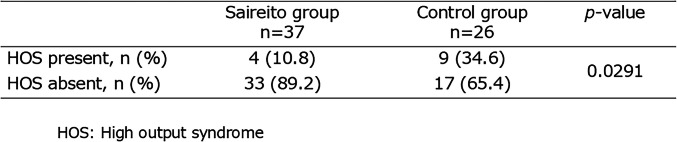
Incidence of high-output syndrome (HOS) in the Saireito and control groups

Among patients who developed HOS, three required intravenous fluid management beyond the scope of routine postoperative care for electrolyte imbalance or dehydration, and outlet obstruction occurred in two patients. The median postoperative length of stay (IQR) was 17 days (15–20) in the HOS group compared with 13 days (10–15) in the non-HOS group, with a statistically significant difference (p = 0.004).

### Other postoperative complications

No significant differences were observed between the two groups in the incidence of postoperative ileus, anastomotic leakage, or outlet obstruction (all p > 0.1) (Table 4). These findings suggest that the administration of Saireito did not increase the risk of postoperative complications. No patient discontinued Saireito because of adverse effects.

**Table 4:**
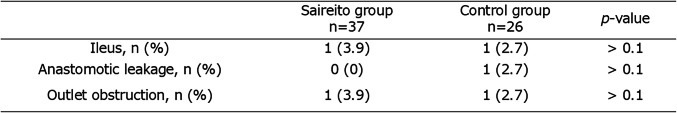
Association between Saireito use and postoperative complications

### Multivariate analysis

Univariate analysis identified intraoperative blood loss ≥ 30 mL (OR, 5.5; 95% CI, 1.104–27.389; p = 0.0374) and non-use of Saireito (OR, 0.229; 95% CI, 0.061–0.853; p = 0.0280) as significant factors associated with the development of HOS.

In multivariate analysis, Saireito administration remained an independent protective factor against HOS (OR, 0.175; 95% CI, 0.038–0.676; p = 0.0108), while blood loss ≥ 30 mL was also independently associated with increased risk (p = 0.0091).

These findings suggest that Saireito use may help reduce the incidence of HOS, defined as stoma output ≥ 1,500 mL/day by POD10 (Table 5).

**Table 5:**
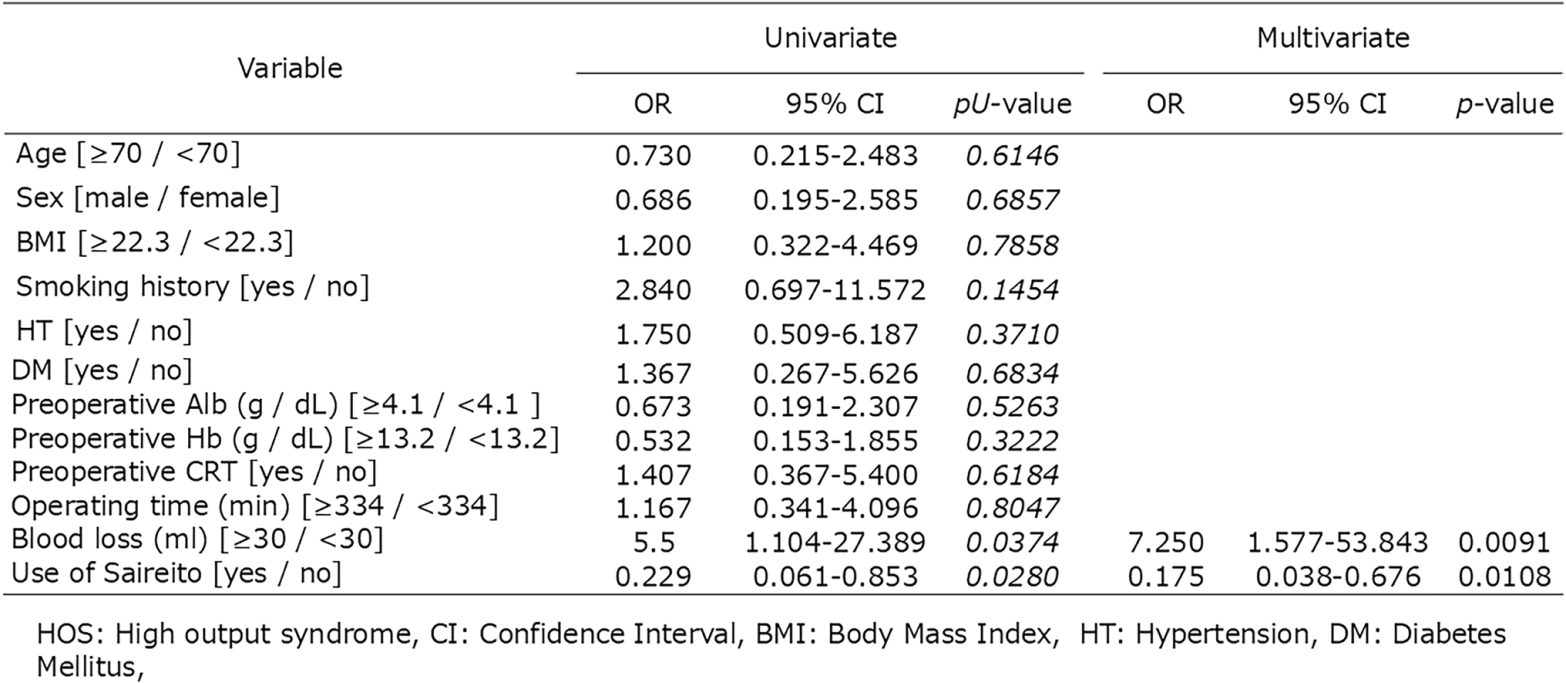
Univariate and multivariate analyses of factors associated with high-output syndrome (HOS)

## Discussion

This study demonstrated that the postoperative administration of Saireito significantly reduced the incidence of HOS in patients who underwent rectal cancer surgery with a temporary ileostomy. Saireito, a traditional Japanese Kampo medicine composed of Shosaikoto and Goreisan, is known for its anti-edema, diuretic, and anti-inflammatory properties. These pharmacologic effects may contribute to the regulation of fluid balance and intestinal secretion, potentially explaining the observed reduction in HOS.

Goreisan exerts a diuretic effect by inhibiting sodium channels in the distal renal tubules. A previous study reported that the diuretic effect of Saireito is approximately 1.4 times greater than that of Goreisan alone in water-loaded mouse models [16]. Furthermore, Shosaikoto may influence the hypothalamic–pituitary–adrenal axis, enhancing endogenous steroid secretion and exhibiting anti-inflammatory and anti-allergic properties [13, 15].

In this study, the incidence of HOS—defined as stoma output ≥ 1,500 mL/day by POD10—was significantly lower in the Saireito group. While no significant differences were observed in other postoperative complications such as ileus, anastomotic leakage, or outlet obstruction, the selective effect of Saireito on HOS prevention is clinically meaningful.

Multivariate analysis identified two independent risk factors for the development of HOS: the absence of Saireito administration and intraoperative blood loss ≥ 30 mL. These findings suggest that surgical factors and perioperative fluid dynamics may interact with intestinal secretion patterns, contributing to HOS onset.

This study has several limitations. First, it was a single-center, retrospective, non-randomized observational study using a historical control group. Although the temporal division of the groups was based on changes in clinical practice, the use of a historical control may introduce observational bias. Second, the evaluation of HOS was limited to the early postoperative period (up to POD10), as accurate measurement of stoma output is not feasible after discharge. Therefore, the effects of Saireito on late-onset HOS could not be assessed. Third, the sample size was relatively small, and no formal sample size calculation was performed because this was a pilot study. The findings should therefore be validated in larger, prospective studies with an appropriate sample size calculation. In addition, the Saireito group included more smokers and tended to include more patients with diabetes than the control group. Although neither factor was significantly associated with HOS in univariate analysis, the potential confounding effect of these background differences cannot be completely excluded.

Nevertheless, HOS remains a significant cause of rehospitalization and postoperative morbidity in clinical practice. The results of this study suggest that Saireito may be a useful strategy for preventing early postoperative HOS. Saireito is approved and reimbursed as a prescription medicine in Japan but is generally not approved for medical use in other countries. Therefore, its international use would require additional clinical trials and regulatory approval processes in each jurisdiction.

## Conclusion

Postoperative administration of Saireito significantly reduced the incidence of high-output syndrome in patients undergoing rectal cancer surgery with temporary ileostomy. Saireito may serve as a safe and effective option for HOS prevention in this setting. Based on these findings, a prospective randomized controlled trial is planned to further validate the preventive effect of Saireito on HOS.

## Supplementary Information

Below is the link to the electronic supplementary material.

Supplementary file1 (TIF 114 KB)

## Data Availability

No datasets were generated or analysed during the current study.
