# Supplementary figures and images for: Saireito (TJ-114) for Preventing High-Output Syndrome After Temporary Ileostomy in Rectal Cancer Surgery
Source: Int J Colorectal Dis. 2025 Oct 6;40(1):209. doi: 10.1007/s00384-025-04983-x (PMC12500839; doi:10.1007/s00384-025-04983-x)

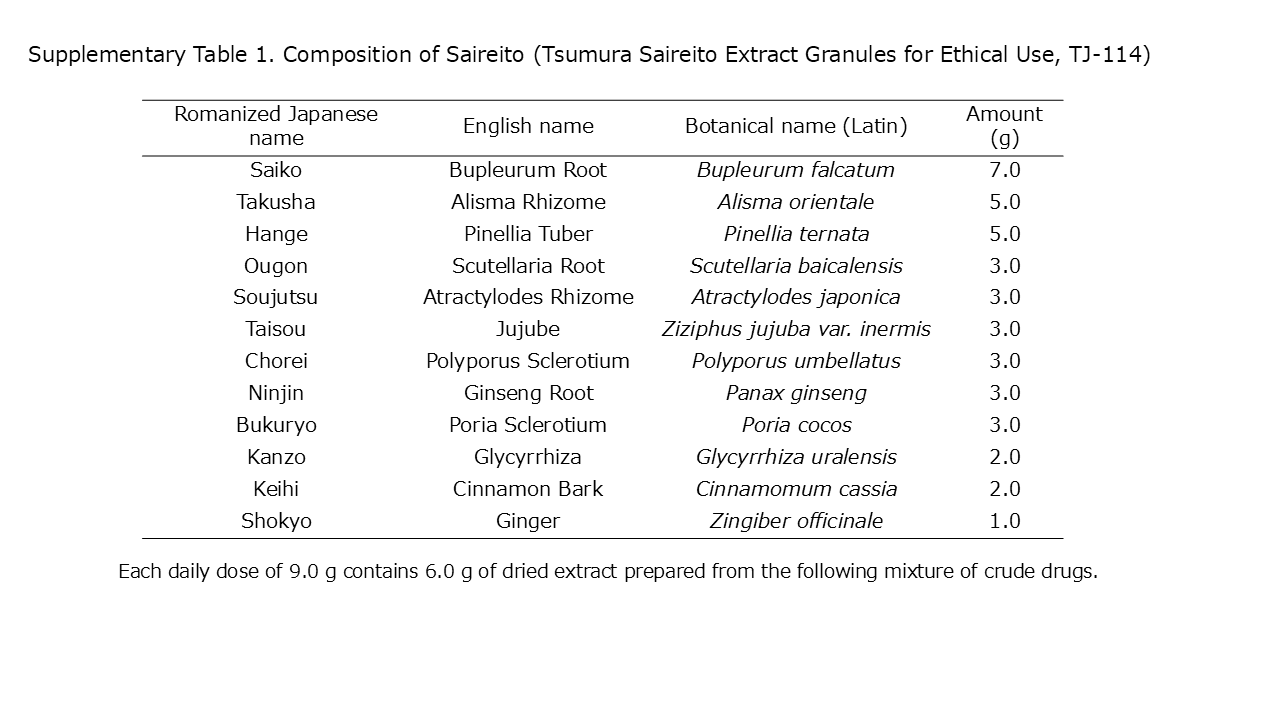

Supplement: Supplementary file 1 — Supplementary file1 (TIF 114 KB) [file 384_2025_4983_MOESM1_ESM.tif]
